# Dazl is a critical player for primordial germ cell formation in medaka

**DOI:** 10.1038/srep28317

**Published:** 2016-06-22

**Authors:** Mingyou Li, Feng Zhu, Zhendong Li, Ni Hong, Yunhan Hong

**Affiliations:** 1 Ministry of Education Key Laboratory of Exploration and Utilization of Aquatic Genetic Resources, College of Fisheries and Life Sciences, Shanghai Ocean University, 999 Hucheng Huan Road, Shanghai 201306, China; 2 Department of Biological Sciences, National University of Singapore, Science Drive 4, Singapore 117543, Singapore; 3 Institute of Bioengineering and Nanotechnology, 31 Biopolis Way, Singapore 138669, Singapore

## Abstract

The DAZ family genes *boule*, *daz* and *dazl* have conserved functions in primordial germ cell (PGC) migration, germ stem cell proliferation, differentiation and meiosis progression. It has remained unknown whether this family is required for PGC formation in developing embryos. Our recent study in the fish medaka (*Oryzias latipes*) has defined dnd as the critical PGC specifier and predicted the presence of additional factors essential for PGC formation. Here we report that *dazl* is a second key player for medaka PGC formation. *Dazl* knockdown did not prevent PGC formation even in the absence of normal somatic structures. It turned out that a high level of Dazl protein was maternally supplied and persisted until gastrulation, and hardly affected by two antisense morpholino oligos targeting the dazl RNA translation. Importantly, microinjection of a Dazl antibody remarkably reduced the number of PGCs and even completely abolished PGC formation without causing detectable somatic abnormality. Therefore, medaka PGC formation requires the Dazl protein as maternal germ plasm component, offering first evidence that dazl is a critical player in PGC formation *in vivo*. Our results demonstrate that antibody neutralization is a powerful tool to study the roles of maternal protein factors in PGC development *in vivo*.

In many animals, the germline is established early in development by primordial germ cell (PGC) specification. PGCs migrate into the gonad, and gonadal germ cells in the adult ovary and testis undergo stem cell self-renewal, differentiation, meiosis and post-meiotic morphogenesis, culminating in the production of eggs and sperm^1^. Defects in any of these processes will lead to reproductive deficiency and infertility. Male infertility represents 40~50% of human infertility and affects one-sixth of couples worldwide^2^,^3^. Male infertility is often associated with azoospermia or oligozoospermia as a consequence of genetic alterations^4^. The DAZ gene family is the best studied that encode infertility factors in animal models^5^ and human^6^,^7^. This family consists of *daz, dazl* and *boule*, which encode RNA-binding proteins that act as functional homologs across phyla^8^,^9^,^10^. The founder member *Daz* is encoded by the human *Deleted-in-azoospermia* and acts as a critical male fertility factor. Four *Daz* genes reside on the human Y chromosome. *Daz* is restricted to primates, its autosomal homolog *Daz*-like (*Dazl*) has been described in several vertebrates including the human^11^,^12^, mouse^13^,^14^, *Xenopus*^15^, axolotl^16^, newt^17^, zebrafish^18^ and medaka^19^. Boule has been considered as the ancestor of the DAZ family and its ortholog has been found in vertebrates^5^,^7^. Boule is the only DAZ family member in invertebrates including *Drosophila*^20^ and *C. elegans*^21^. In the rainbow trout, differential expression of *boule* and *dazl* reveals germ cell sex prior to meiosis^22^,^23^.

The DAZ family is exclusively required for germ cell development. The functions of the family members are, however, distinct and variable in different organisms. In invertebrates, *boule* is expressed only in the ovary and required for oogenesis of *C. elegans*^21^, but is essential for meiotic cell cycle in spermatogenesis of *Drosophila*, as male mutants are sterile and their spermatocytes are arrested at the G2/M phase^20^,^24^. In vertebrates, *dazl* depletion in *Xenopus* leads to defective PGC development^8^, targeted *dazl* disruption in mouse results in the sterility in both sexes, with the prime spermatogenic defect being a failure of spermatogonial differentiation as germ cells in the testis are arrested at spermatogonial stage^14^. Several studies *in vitro* have revealed a role of the DAZ family members in germ cell fate decision. In mouse ES cells in culture, forced *dazl* expression promotes germ cell formation^25^. In human ES cells, Dazl functions also in germ cell formation, whereas Daz and Boule promote later stages of meiosis and development of haploid gametes^26^. It has remained unknown whether Dazl functions PGC specification in developing embryos.

Diverse animal species make use of two distinct modes for PGC formation, namely preformation and epigenesis^27^,^28^. Preformation operates in egg-laying animals such as *Drosophila*^29^, *C. elegans*^30^ and *Xenopus*^15^. In these organisms, the cytoplasmic germ plasm is maternally supplied to the embryo, asymmetrically partitioned to one or few cells to intrinsically determine the PGC fate before or during cleavage divisions. Epigenesis prevails in mammals such as mouse^31^ and urodelean amphibians such as newt^16^. In epigenesis, maternal inheritance of germ plasm components is absent, and PGC formation is independent of germ plasm but extrinsically induced by cell-cell interactions during gastrulation^31^. In fish, PGC preformation has been demonstrated in zebrafish and medaka. In zebrafish, germ plasm components are maternally inherited and asymmetrically segregated into pPGCs during early cleavages^32^,^33^,^34^. In medaka (*Oryzias latipes*), embryo perturbation does not affect the PGC number, leading to the notion for PGC preformation in this organism^35^. Unusually, medaka maternal germ plasm components, such as the transcripts of *boule* and *dazl*^5^,^19^*, vasa*^36^,^37^,^38^ and *piwi*^9^, distribute widely during early development rather than localization into a small number of cells. In addition, knockdown of germ genes such as *nanos*^39^, *vasa*^36^ or *piwi*^9^ can reduce the number of PGCs and affect PGC migration but cannot completely preventing PGC formation. Direct evidence for medaka PGC preformation comes from the observation that associated single cells from midblastula embryos in culture are able to form PGCs in the absence of normal somatic structures and known inducing factors^40^. Most recently, we have identified *dnd* as the critical PGC specifier in medaka and predicted the presence of additional factors essential for PGC formation, as dnd overexpression can enhance the PGC number by up to 3 folds, and many *dnd*-expressing cells adopt somatic cell fates^41^. This study was aimed at analyzing the role of *dazl* in medaka PGC development. We show that injection of an anti-Dazl antibody is able to abolish PGC formation in medaka embryos, providing first evidence that maternal Dazl is required for PGC formation *in vivo*.

## Results

### Effect of *dazl* knockdown on PGC development

Transgenic medaka lines Ng and Vg were used for PGC observation, which express GFP from the medaka *nanos3* and *vasa* promoter (*olvas-gfp*) exclusively in germ cells, respectively^36^. To trace PGCs specifically by zygotic GFP expression, hybrid embryos (referred NgVg embryos thereafter) between Ng females and Vg males were produced for monitoring PGC development^36^.

**Figure 1:**
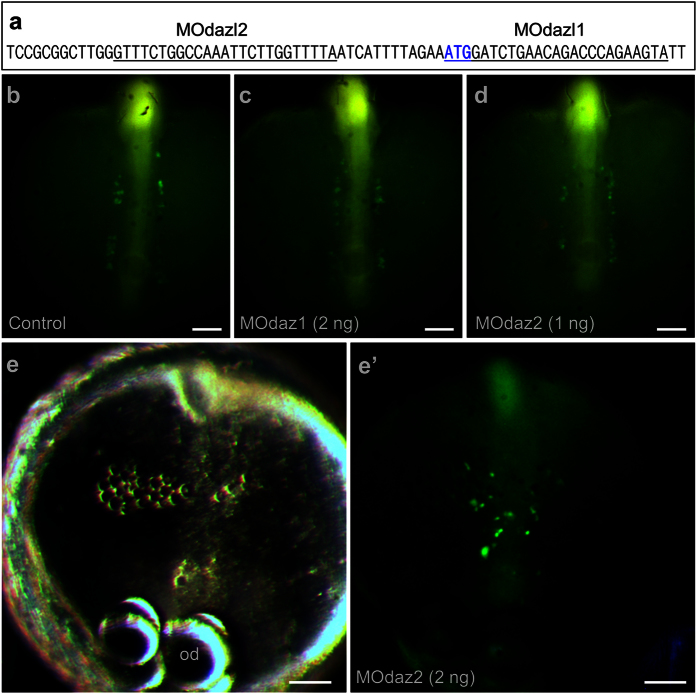
dazl knockdown has little effect on PGC formation. NgVg embryos were injected at the 1-cell stage and analyzed microscopically at stage 22 for PGCs (green). (**a**) Positions of MOdaz1 and MOdaz2. The target sequences on the medaka dazl cDNA are underlined. The ATG codon is shown in bold. (**b–d**) Normal somatic development and PGC formation after injection with water (**b**), MOdaz1 (**c**) and MOdaz2 (**d**). (**e**,**e’**) Abnormal somatic development and normal PGC formation after morpholino injection. The anterior is to the top. od, oil droplet. Scale bars, 100 μm.

Several experiments have suggested that medaka PGC formation is independent on somatic development^9^,^35^,^36^. In zebrafish, microinjection of antisense morpholino oligos against germ plasm RNA components such as *vasa*^42^, *nanos*^43^ and *dnd*^44^ does not affect PGC formation. In medaka, microinjection of morpholinos against *vasa*^45^ and *piwi*^9^ affects PGC migration but does not prevent PGC formation. Most recently, we show that *dnd* acts as the medaka PGC specifier^41^. We extended our study to *dazl* for analyzing the role of a maternal factor in medaka PGC formation. The *dazl* RNA is a maternally supplied germ plasm component in medaka^19^. In mouse, forced *dazl* expression promotes germ cell formation from ES cells in culture^25^. In human, Dazl functions in germ cell formation from ES cells, whereas closely related genes Daz and Boule promote later stages of meiosis and development of haploid gametes^26^. Two series of experiments were performed. To this end, NgVg embryos at the 2-cell stage were subjected to microinjection of antisense morpholino oligos (MOs) against the medaka *dazl*. Two MOs were used: MOdaz1 targets the sequence spanning the ATG codon, MOdaz2 recognizes the sequence upstream of the ATG (Fig. 1a). Microinjection of MOdaz1 at 2 ng or MOdaz2 at 1 ng was permissive for normal somatic development and PGC formation (Fig. 1b–d). MO injection at higher doses, namely MOdaz2 at 2 ng prevented somatic development, resulting in a disorganized cell mass that lacked normal embryonic structures. Interestingly, even in these severely disorganized embryos, PGC formation was not prevented (Fig. 1e,e’). Similarly, coinjection of MOdaz1 and MOdaz2 at 1 ng led to abnormal somatic development and seemingly normal PGC formation (Figure S1). In a total of 83 MO-injected embryos, we failed to detect a remarkable reduction in the number of PGCs. As summarized in Table 1, a control embryo after water injection produces 31.8 PGCs at 40 hpf, which is not significantly different from 29.3 of those injected with MOdaz1 and 33.7 of those injected with MOdaz2. Although it is unclear whether abnormal somatic development is due to the toxicity of MOdaz1 and MOdaz2 or an essential role of *dazl* in somatic development of early medaka embryos, these data demonstrate that medaka PGCs can form in the absence of a normal somatic environment, conforming to the preformation mode in this organism.

### Embryonic Dazl protein expression

**Figure 2:**
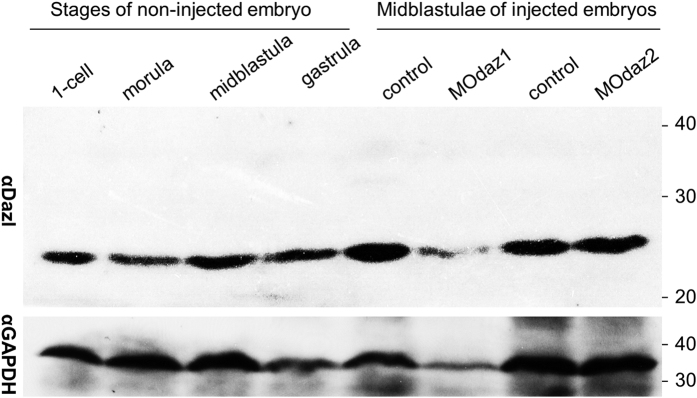
Western blot analysis of Dazl protein expression. Crude protein extract from three embryos was used for each lane. GAPDH was detected as a loading control. Size markers in kilodalton are shown to the right. MOdaz1 and MOdaz2 were injected at 2 ng and 1 ng to the1-cell embryos, respectively.

Injection of *dazl* MOs affects the soma but not PGC formation in medaka embryos is unusual, because *dazl* is sufficient to promote germ cell formation from mammalian ES cells^25^,^26^. Since MOs act through the inhibition of translation, we performed a Western analysis on Dazl protein expression in developing medaka embryos by using αDazl, a polyclonal anti-Dazl antiserum capable of specifically staining medaka germ cells in the adult testis and ovary^19^. The Dazl protein was seen at a high level already in 1-cell embryos and until gastrulation, and this level was not reduced significantly by MOdaz1 or MOdaz2 (Fig. 2). Therefore, the Dazl protein in medaka is maternally supplied at a high level and persists until gastrulation when PGC formation occurs, and it is not surprising that *dazl* MOs are inefficient to reduce the Dazl level and thus unable to exhibit effect on PGC development in this organism.

### Medaka PGC development requires maternal Dazl

**Figure 3:**
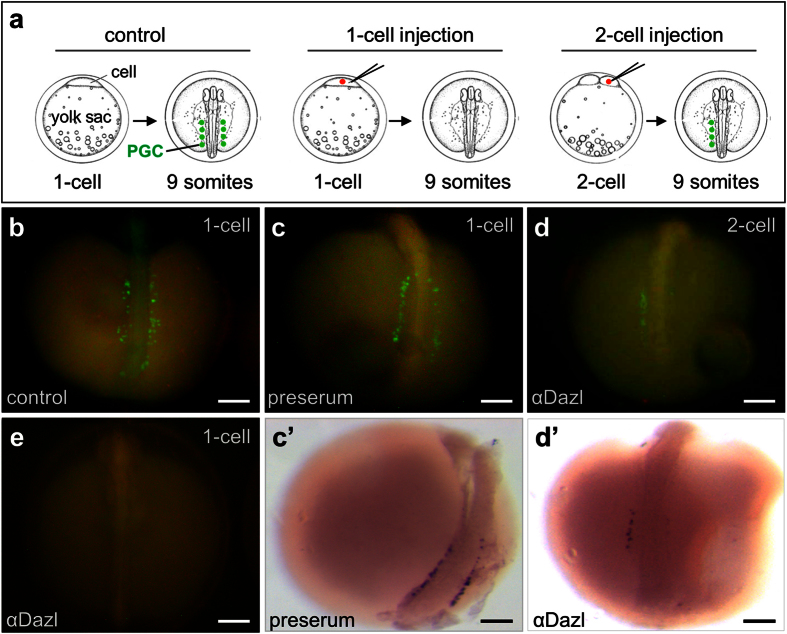
Dazl is required for medaka PGC formation. Vg embryos were monitored for PGCs by GFP expression and *in situ* hybridization at 40 hpf. (**a**) Schematic microinjection at the 1-cell or 2-cell stage and PGC detection at the 9-somite stage. (**b**,**c**) Control Vg embryos, showing many PGCs (green) in two bilateral clusters along the embryo axis without (**b**) or with preserum injection. (**d**) Embryo after αDazl injection at the 2-cell stage, showing the absence of PGCs in the right side from the injected cell. (**e**) Embryo after αDazl injection at the 1-cell stage, showing the absence of PGCs. (**c’**,**d’**) Embryos shown in (**c,d**) after *in situ* hybridization with an antisense dazl riboprobe, showing the presence of ~34 PGCs on both sides (**c**’) and only 9 PGCs in the left side from the non-injected cell (**d**’). 1-cell, microinjection at the 1-cell stage; 2-cell, microinjection into one of the 2 cells at the 2-cell stage. Scale bars, 100 μm.

In *Xenopus*, injection of an anti-Vasa antibody perturbed the function resulted in failure of PGC differentiation at the tadpole stage^46^. The inefficiency of *dazl* MOs in reducing the Dazl protein level due to an abundant maternal supply provoked αDazl injection to neutralize the Dazl activity. To this end, NgVg embryos at the 2-cell stage were injected with αDazl or preserum as a control. When injected at high doses (5~10 ng per embryo), either antibody produced abnormal embryos. Upon injection with either antibody at 3 ng per embryo, the majority of embryos appeared normal. As expected, injection of water and a preserum did not affect PGC formation (Fig. 3a,b), and produced an average of 33.5 PGCs among 25 embryos at 2 dpf. A total of 52 embryos at the 1-cell stage were injected with αDazl, 43 survivors at 2 dpf exhibited seemingly normal development and were analyzed for PGC development. This revealed that the average PGC number decreased by 30.5% to 23.3 (Table 2). The inhibitory effect of αDazl on the PGC number became more evident when PGCs were examined for bilaterally asymmetric distribution. In controls, averages of PGCs were 14.5 and 19.0 at the left and right sides, respectively. These values became 7.9 and 15.4 in αDazl-injected embryos, giving rise to a reduction by 45.5% and 18.9%, respectively (Table 2). Upon αDazl injection at the 2-cell stage, the absence of PGCs on the side from the injected blastomeres was seen in certain Vg embryos (Fig. 3c). Most convincingly, two of the 43 Vg embryos injected with αDazl at the 1-cell stage were found to be completely free of PGCs (Fig. 3d). *In situ* hybridization by using an antisense *dazl* riboprobe revealed the presence of ~34 PGCs in preserum-injected control embryo (Fig. 3b’) but only 9 PGCs upon αDazl injection (Fig. 3c’). Collectively, medaka PGC formation requires the maternal Dazl protein.

## Discussion

The transcripts and protein products of germ genes are often germ plasm components that are maternally supplied in many egg-laying organisms. In zebrafish, maternal RNA inheritance has been known for *vasa*^32^,^34^, *nanos*^43^, *dnd*^44^, *zili*^47^ and *ziwi*^48^, and microinjection of antisense morpholino oligos against some of them including *nanos*^43^ and *dnd*^44^ leads to abnormal PGC development, ranging from a reduced PGC number over defective PGC migration to survival. In medaka, maternal RNA inheritance has been reported for *vasa* and *piwi*, and microinjection of their antisense morpholino oligos results in a reduced PGC number and defective PGC migration^9^,^36^. Interestingly, accumulated data in fish show that antisense morpholino oligos of germ genes cannot completely prevent PGC formation but merely affect subsequent steps of PGC development, compared to their requirement for PGC formation as illustrated by *vasa* loss-of-function mutations in *Drosophila*^46^. A difference in phenotype between morpholino-mediated translation inhibition and loss-of-function mutations has been ascribed to a high level of maternal protein supply in combination with incomplete translation inhibition^36^. In this study, we provide first evidence in medaka that the Dazl protein is indeed maternally supplied at a high level and persists until gastrulation when PGC formation occurs. Consequently, *dazl* morpholino oligos have little effect on the Dazl protein level and thus on PGC development. We demonstrate that αDazl injection is sufficient to remarkably reduce the PGC number and even to abolish PGC formation in certain cases, perhaps via neutralizing the activity of Dazl protein. Our data suggest that antibody injection offers an alternative tool to study the earliest event of PGC development, namely PGC formation in fish, as has been reported in *Xenopus*^49^.

In this study, we have revealed that αDazl injection leads to severe reduction in the PGC number and even a complete loss of PGCs, demonstrating that dazl plays an essential role in PGC formation. The PGC absence may be due to the absence of PGC formation or disappearance of PGCs by death prior to observation. Three observations favor the absence of PGC formation in certain αDazl-injected embryos. First, zebrafish PGC survival requires the function of *nanos*^43^ and *dnd*^44^, and PGCs are visible by transient GFP expression during somitogenesis and begin to die afterwards upon nanos or dnd knockdown. Second, vasa or piwi knockdown in medaka does not affect PGC survival even at ectopic sites of advanced embryos or in culture^9^,^36^. Finally, GFP or RFP is fairly stable and its fluorescence can persist in dead cells for 3 days, as illustrated by cell culture in the presence of puromycin^40^. Medaka PGCs are visible by transient GFP expression until 13 hpf^39^, and PGC observation in this study has been made from 40 hpf onwards. Well-specified PGCs, either live or dead, should be identifiable by GFP expression, suggesting that observation in medaka at 40 hpf is able to detect the majority–if not all–of previously formed PGCs. The fact that αDazl injection leads to a reduction or even loss of PGCs suggests a role for *dazl* in medaka PGC formation. Previously, we have shown in medaka that *vasa* or *piwi* knockdown reduces the PGC number^9^,^36^. Most recently, we have identified dnd as the critical PGC specifier and predicted the presence of additional factors in medaka PGC formation^41^. Results in this study reveals *dazl* as the second key player in PGC formation. In mammals, forced *dazl* expression *in vitro* promotes germ cell formation from ES cells of mouse^25^ and human^26^. Hence, *dazl* plays a conserved role for PGC development from fish to mammals.

## Materials and Methods

### Animals

Work with animals was carried out in strict accordance with the recommendations in the Guide for the Care and Use of Laboratory Animals of the National Advisory Committee for Laboratory Animal Research in Singapore and approved by this committee (Permit Number: 27/09). Medaka strains HB32C and af were maintained under an artificial photoperiod of 14-h light to 10-h darkness at 26 °C^50^,^51^,^52^. Transgenic line Vg was described previously^51^, which expresses GFP from the medaka *vasa* promoter^36^. Heterozygous Vg embryos were produced by crossing homozygous Vg males to non-transgenic females and used for microinjection and cell culture. In certain experiments, heterozygous Vg males were crossed with non-transgenic females, and resultant embryos were used for cell cultures.

### Morpholino oligos

Morpholino antisense oligos were purchased from Gene Tools (Oregon) and dissolved in water. MOdaz1 (TACTTCTGGGTCTGTTCAGATCCAT) and MOdaz2 (TAAAACCAAGAATTTGGCCAGAAAC) target the medaka *dazl* RNA (Accession number AY973274), the former spans the initiation codon (underlined), and latter is positioned 12 nt upstream of the initiation codon.

### Antibodies

Control preserum and polyclonal anti-Dazl antisera (αDazl) were produced and used as previously described^19^.

### Embryo injections

Embryos were injected at the 1- or 2-cell stages as described^36^. MOdaz1 and MOdaz2 were dissolved at 0.1~5 mg/ml, which corresponds to 0.1~5 ng per injection. Preserum and αDazl were diluted in water at 1:3 before injection, corresponding to 3 ng protein per embryo as determined by using the BioRad protein assay kit (#500-0006). Successful injection was monitored on the basis of co-injected fluorescent dye Texas red.

### *In situ* hybridization

Embryos were fixed and subjected to *in situ* hybridization with an antisense *dazl* riboprobe as described^5^,^19^.

### Western blot analysis

Homogenates of whole embryos at representative stages were resolved in 10% SDS-PAGE and blotted as described (Xu *et al*., 2005). The blots were incubated with αDazl or αGAPDH, the latter being a monoclonal mouse antibody against the human glyceraldehyde-3-phosphate dehydrogenase (GAPDH) at a 1:1000 dilution (sc-47724, Santa Cruz Biotechnology, Inc.). After washing, the blots were incubated with secondary antibodies (A0545 or A9044, Sigma) at a 10,000 dilution and visualized by the ECL detection reagents (Pierce, USA).

### Microscopy

Microscopy was done as described^5^,^50^,^53^. Briefly, live embryos and fry were visualized using a Leica MZFLIII stereo microscope equipped with a Fluo III UV-light system and a GFP2 filter and photographed by using a Nikon E4500 digital camera (Nikon Corp). For documentation at larger magnification, live embryos and fry were observed and photographed on Zeiss Axiovert2 invert microscope equipped with a Zeiss AxioCam MRc digital camera and AxioVision 4 software.

### Statistics

Statistical analyses were calculated by using GraphPad Prism v4.0. Data consolidated were presented as mean ± s.d.

## Additional Information

**How to cite this article**: Li, M. *et al*. Dazl is a critical player for primordial germ cell formation in medaka. *Sci. Rep.* **6**, 28317; doi: 10.1038/srep28317 (2016).

## Supplementary Material

Supplementary Information

## Figures and Tables

**Table 1:** Effect of *dazl* morpholinos on the PGC number^1^.

Injection	dose	Number of embryos observed	Number of PGCs per embryo^2^
water		52	31.8 ± 5.5
MOdaz1	2 ng	47	29.3 ± 7.4
MOdaz2	1 ng	36	33.7 ± 8.0

^1^NgVg embryos were injected at the 1-cell stage. PGCs were scored by GFP expression at 40 hpf.

^2^Data are mean ± s.d. No significant difference was observed between water injection control and experimental groups injected with MOdaz1 or MOdaz2.

**Table 2:** Dazl depletion blocks PGC formation^1^.

Serum injected	Number of embryos	Number of PGCs		
		Total	Side 1^2^	Side 2^2^
Preserum	25	33.5 ± 5.3	14.5 ± 3.2	19.0 ± 3.1
αDazl	43	23.3 ± 6.4	7.9 ± 3.5	15.4 ± 4.6

^1^Preserum or αDazl was injected with 1.5 ng per NgVg embryo into one of the two cells at the 2-cell stage, and PGCs were scored by GFP expression at 40 hpf.

^2^Side 1 is the injected side, which was labeled by a co-injected fluorescent dye, and side 2 is the noninjected side. Significant difference was observed between preserum injection and αDazl injection in the injected side but not noninjected side.
