# Supplementary material for: Dazl is a critical player for primordial germ cell formation in medaka
Source: Sci Rep. 2016 Jun 22;6:28317. doi: 10.1038/srep28317 (PMC4916430; doi:10.1038/srep28317)
Supplement: Supplementary Information [file srep28317-s1.pdf]

Dazl is a critical player for primordial germ cell formation in medaka

Mingyou Li, Feng Zhu, Zhendong Li, Ni Hong, Yunhan Hong*

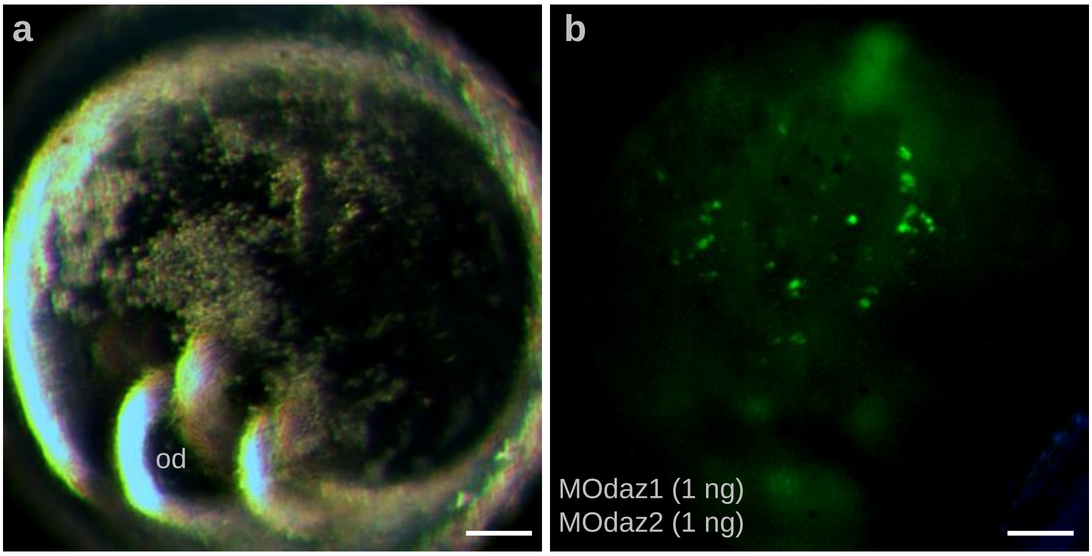

Figure S1 | dazl knockdown does not affect PGC formation. NgVg embryos were injected at the 1-cell stage with 1 ng of MOdaz1 and 1 ng of MOdaz2 for microscopic analysis at stage 22. **a** and **b**, Micrographs at bright field (a) and fluorescent optics (b), showing abnormal somatic development and a normal PGC number. The anterior is to the top. od, oil droplet. Scale bars, 100 µm.
